# High school students’ knowledge and experience with a peer who committed or attempted suicide: a focus group study

**DOI:** 10.1186/1471-2458-14-1081

**Published:** 2014-10-18

**Authors:** Hilda N Shilubane, Robert AC Ruiter, Arjan ER Bos, Priscilla S Reddy, Bart van den Borne

**Affiliations:** Department of Advanced Nursing Sciences, University of Venda, Private Bag X 5050, Thohoyandou, South Africa; Department of Work and Social Psychology, Maastricht University, Maastricht, The Netherlands; Department of Clinical Psychology, Open University, Limburg, The Netherlands; Population Health, Health Systems and Innovations (PHHSI) Research Programme, Human Sciences Research Council, Cape Town, South Africa; Department of Health Promotion, Maastricht University, Maastricht, The Netherlands

## Abstract

**Background:**

Suicide is a major public health problem for adolescents in South Africa, and also affects those associated with them. Peers become more important during adolescence and can be a significant source of social support. Because peers may be the first to notice psychological problems among each other, the present study’s objectives were to assess students’ knowledge about suicide, perceived risk factors, signs of poor mental health in adolescents who committed suicide, students’ awareness of available mental health care and resources, and beliefs about prevention.

**Methods:**

This qualitative study used focus group discussions to elicit the thoughts and feelings of high school students who had a peer who committed or attempted suicide. Peers and class mates of suicide attempters and suicide completers were identified with the help of a social worker and school management and were invited to participate. All focus group discussions were audio taped and analyzed. A total of 56 adolescents (13–19 years of age) from Limpopo schools in South Africa participated in six focus group discussions. The data were analyzed by NVivo version 8, using an inductive approach.

**Results:**

Participants reported to be affected by the suicide attempt or completed suicide. They felt guilty about their failure to identify and prevent the suicide and displayed little knowledge of warning signs for suicidal behaviour. They identified several risk factors for the suicide of their peers, such as poor relationship issues, teenage pregnancy, punishment, and attention seeking behaviour. Resources for students with mental health problems and survivors of suicide attempts were not perceived to be available at schools and elsewhere.

**Conclusion:**

School-based suicide prevention programs based on theory and evidence are necessary. Such interventions should also focus on detection of mental health problems by peers. Counseling services for students with mental health problems and suicide survivors should be available and made known to students at risk and peers.

## Background

Suicide is a complex public health problem with psychological, social, biological, cultural and environmental factors involved [1–7]. Suicide rates in Limpopo (South Africa) are very high, especially among adolescents [6]. During adolescence, peers become more important and are among the first to notice mental health problems in other youngsters. Supportive peer relationships are important for adolescents as they promote their social, emotional and relational well-being, whereas having poor peer relationships is associated with suicide ideation and suicide attempts (Fotti, Katz, Afifi, & Cox, [8]; Cohen & Prinstein, [9]; Kaminski & Fang, [10]; Kerr, Preuss, & King, [11]; Walker & Greene, [12]). However, Kerr, Preuss and King [11] also found that greater levels of perceived peer support were associated with higher levels of hopelessness, depressive symptoms, and suicidal ideation among males. It is therefore possible that suicidal youth affiliate with and are negatively influenced by depressed and suicidal peers, but having friends can also be a protective factor to suicide attempts for both girls and boys [8, 13]. Research has largely neglected the role of peers in the prevention of adolescent suicide. The present study examines experiences of high school students whose peer attempted or committed suicide and tries to shed additional light on the role of peers in suicide prevention.

The first and second South Africa National Youth Risk Behaviour Survey in 2002 and 2008, respectively, found that 17.3% and 21.4% of adolescents made one or more suicide attempts in the past six months with no significant differences by gender [5, 6]. In the study of 2008, Limpopo province was found to have the highest rate of suicide attempts (24.5%) [6]. This province is home for 5 404 868 people (10.4% of the total population of South Africa) and just over 40% of the population are younger than 15 years old [14].

Several studies have been conducted on the psychosocial determinants of suicide ideation and suicide attempts, also in developing countries. For example, Peltzer and Pengpid [15] in their study among adolescents in Thailand found that sadness, lack of parental attachment, current alcohol use, and ever having had sexual intercourse were found to influence suicidal ideation. In a qualitative study among adolescents in Limpopo, Shilubane and colleagues [16] found that lack of knowledge of available counselors, conflict in interpersonal relationships, perceived accusations of negative behaviour, inadequate social support, past family and peer suicide attempts, and poor living circumstances were factors related to suicide attempts. A subsequent quantitative study among the same population demonstrated that suicide ideation is prevalent among these adolescents, and psychosocial factors of perceived social support and negative feelings about the family and behavioural factors of forced sexual intercourse, and physical violence by the partner increased the risk of suicidal ideation. This study also showed that depression mediated the relationship between the psychosocial and behavioural risk factors and suicide ideation [17]. Peltzer et al. [18] examined the correlates of suicide risk among secondary students in Cape Town. Their study revealed that anger control problems, low self-esteem, perceived stress and unmet school goals were identified as predictors for suicide risk.

Other studies have examined the role of family and parents in adolescent suicide prevention [19–23], but few have focused on the role of peer relationships in suicide in schools. Most adolescents are enrolled in schools and this environment provides adolescents with meaningful relationships with peers that may have important consequences for adolescent development and coping with problems [24]. Supportive peer relationships are important for adolescents as they promote adolescents’ social, emotional and relational well-being, whereas having poor peer relationships is associated with suicide ideation and attempts [8–12].

In the lives of high school students peers are among the first to know of a student’s mental health problems or suicidal thoughts, but peers are also frequently unsure what to do with this information [25]. In many instances, peers are the most important source of social support. Peers could thus be an important target group for suicide prevention programs targeting adolescents. The present study was conducted to describe the impact on high school students of a suicide or suicide attempt by a peer, to assess students’ knowledge about suicide, perceived risk factors, signs of poor mental health, and to assess their awareness of available mental health care and resources and opinions on prevention.

## Methods

### Design and participants

Six focus group discussions were conducted, using an in-depth focus-group technique. Study participants consisted of 56 high school, Xitsonga speaking students who lived in Limpopo province, South Africa (SA). Groups were of mixed gender, totalling 30 girls and 26 boys. Two focus groups with high school students whose peer attempted suicide and four focus groups with students whose peer completed suicide were conducted. All focus groups were conducted in different schools. Each focus group consisted of eight to ten participants and was facilitated by the first author.

### Sampling procedure

Regarding the selection of suicide attempters, the first author contacted the social worker at the hospital and requested her to ask the student who attempted a suicide during counseling sessions if they were willing to be contacted by the first author to discuss their suicide attempts. A list of attempters who agreed to be contacted was given to the first author by the social worker. The first author wrote names of the attempters on pieces of paper, which were placed in a bowl. The first author then randomly picked out two pieces of papers with names of attempters. The first author then contacted the students who attempted a suicide and asked if they were willing to allow their peers or class mates to discuss about their experiences of the suicide attempt. She asked the suicide attempters for names and telephone numbers of their peers or class mates to be contacted for a group interview. The first author contacted these peers or class mates and explained the aim of the project, and asked if they were willing to take part while informing them that their class mate had given his or her consent. The time and place for the discussions was then communicated to participants.

The peers in the focus groups of suicide completers were selected in the following manner. During the focus group discussions with the peers of suicide attempters in one of the groups the name of a student from another school who recently completed suicide was mentioned. Thus the researcher visited the school where the student schooled to ask for permission to conduct a focus group discussion with the class mates of the student. A snow ball procedure was then used to find other schools that recently lost a student through a suicide. After talking to the school principal and agreement of the school principal, at each school a teacher was assigned by the principal to assist the first author. The first author recruited participants who were peers and class mates of the deceased. Those who consented were invited for a focus group meeting. The same interview schedule was used for each focus group although this was used as a flexible guide rather than a rigid protocol. Focus group discussions were held at schools at a time convenient to the individual participants and the host organizations.

### The focus group procedure

The first author, a professional nurse, moderated the focus groups. At the beginning of each session, the moderator introduced the project and explained the purpose of the focus group. The opening question posed to students was: “How often do you think about your peer who attempted (or completed) suicide”. The interview guide was used as a flexible tool to guide the discussions which were audio-recorded. The length of the discussions ranged from 60–90 minutes. Participants were informed again that participation was voluntary and that they were free to withdraw from participation at any time without penalties. All participants agreed to participate in the study and none withdrew before the end of the focus group session. The total of six focus group discussions proved to be sufficient to reach data saturation.

### The interview schedule

The interview schedule was translated and used in the local language of the participants (Xitsonga). The selection of research themes for the focus group discussions was based on a recent qualitative study on suicide attempts among black South African adolescents [16] and a quantitative study on psychosocial correlates of suicide ideation in South African adolescents [26] together with issues derived from the literature. Key themes were feelings and emotional effects of the suicide attempt or completed suicide of their peer, students’ knowledge about suicide, perceived risk factors, signs of poor mental health, students’ awareness of available mental health care and resources for prevention, and opinions on suicide prevention.

### Ethical consent

The Ethics Committee of the University of Venda provided approval for the study. Permission to conduct the study was obtained from the provincial Department of Health, Department of Education, Chief Executive Officer of participating hospital, and principals of schools. The participants were informed that participation was voluntary and that confidentiality of their information would be ensured. Permission and written consent was also obtained from participants prior to interviewing and voice recording of the focus group interviews. The participants were assured that their responses would not be linked to their personal identities and that after transcription the recordings would be destroyed afterwards. They were informed at the beginning that should they need counseling as a result of the participation in the study, arrangements were made with a private counselor. None of the participants sought counseling as a result of their participation in the focus group discussions. In fact, at the end of the discussions participants indicated that they had valued the experience and opportunity to discuss issues surrounding the suicide or suicide attempt of their peer in a safe environment.

### Data analysis

Immediately after each group discussion, the data were transcribed in Xitsonga and translated in English by a first language Xitsonga speaker. The accuracy of the translations was checked randomly by a second first language Xitsonga speaking research assistant. The English and the Xitsonga versions of the interviews were then carefully read and checked by the first author as she had conducted the interviews and is fluent in both Xitsonga and English. The data were analysed by using NVivo version 8, using a general inductive approach. The predefined themes were explored and an inductive process was used to derive subthemes from the main themes. As coding occurred, a ‘tree structure’ was generated in which themes and subthemes were linked to one another. The findings below are structured according to these themes and subthemes. RATS guideline for reporting qualitative studies were adhered at all points during the study.

## Results

### Method used to attempt and commit suicide

The following methods were used by victims for their suicide attempts or completed suicide, two attempters took overdose of tablets. The four suicide victims used hanging to kill themselves.

### Reactions of students

Participants whose peer completed suicide reported that they were affected emotionally by the student’s suicide. Some cried after hearing the death, some wished for the supernatural powers to be able to communicate with the deceased, while others became preoccupied with suicidal thoughts. A female peer at a private school said: “*It caused us much pain. I was personally pained. It took me away from that point; in fact I am not sure as to whether it is a point or something else. But then I remember one day in my room before I slept I prayed. I prayed to God to give me the power and ability to communicate with ghosts so that I can talk to him and can pass messages through to others”*. Another student said: “*I remember the other day, I think it was the day of the funeral or memorial service, he said: ‘this week you will bury my friend and next week you will bury me”.*

The majority of the students blamed themselves for the peer’s suicide. The self-blame was attributed to several issues. Some blamed themselves for failing to probe deeper when the peer mentioned that he was tired to live. *“I also felt guilty because there was a time he wanted to be my friend but I was not interested in making the friendship with him. When I learned about his death I thought maybe I did something that made him to think that I hate him, and then felt lonely after I refused to be his friend and this made me feel bad”* (female friend at a private school). Other peers blamed themselves because the victim at some stage initiated friendship with them, but they declined and after hearing about the suicide they concluded that their unwillingness to make friendship made the deceased feel lonely and unwanted while some participants blamed themselves for failing to identify that the peer had problems, and wished they could have identified it in order to have offered help. They thought that they might be the persons who contributed to the suicide since they shared most of their life experiences with the deceased, thus adding pain to the already existing ones as evidenced by this quote *“I blame myself because we used to share a lot of things. I think I could have added salt unto any injury unknowingly. I don’t know what caused her not to confide in me until she did such a horrible thing”* (female classmate).

In the focus group discussions with peers of attempters, similar emotions were felt, although the psychological pain seemed less intense. The suicide attempt of the peer brought back the memories of a friend they lost some years ago. The students were desperate, because they were not sure of the outcome of the attempt, and were afraid that they were going to lose a second friend within a short space of time. “*I personally was affected because she was going to be a second friend to loose without knowing the cause. I was going to have a problem since I depend on her as ‘the only surviving friend since the other friend passed on while we were doing grade 11”,* said a female student.

The group of students reacted differently to the two situations. Those that lost a peer appeared to have experienced more psychological pain than the group whose friend survived the suicide attempt.

### Signs of poor mental health

Participants who lost a peer by suicide reported that the deceased peers displayed unusual behaviour some weeks or days before their suicide. There were changes in behaviours, such as sleeping during lessons, talking about death, withdrawing from social interactions, exhibiting mood changes, and writing messages on ‘mixit’ (a mobile instant messenger application developed in SA that runs on GPRS/3G mobile phones and allows you to communicate with other ‘mixit’ users on their cell phones). The following quote shows the message sent to friends three days before the peer commit suicide. *“I will die for my friends”*. He then listed the names of some of his friends.

The peers observed different behaviours of the suicide attempters and suicide completers before the attempt or suicide. The group of students with a peer who attempted suicide reported that they did not observe anything unusual and that the victim was happy and behaved like the previous days, like doing her school work and never slept during the lessons. Contrary, the completers group observed strange behaviour from peers before they died. The behaviour made them conclude after their death that they bid their peers farewell.

### Perceived cause of the peer’s suicide attempt/suicide

Views on the perceived cause of the peer’s suicide varied. The causes that were perceived by the students with a peer who completed suicide were related to teenage pregnancy, punishment, attention seeking behaviour, and curse. In two groups the students viewed teenage pregnancy to have caused their peer to commit suicide. They thought the student could have been fallen pregnant and was afraid to stand the pressure of fellow male students who had a tendency of asking the teacher uncomfortable questions during lessons. They thought since the student was a quiet person and regarded herself a child of God, people were going to mock at her. *“As she was a girl she could have fallen pregnant and might have thought that the boys, more especially, would have laughed at her. Also there is a tendency of asking uncomfortable questions when the teacher is in class regarding the issue”,* said a female student.

Participants in one focus group mentioned that the Deputy Principal gave a student a punishment for failing to complete an assignment that was overdue. The participants thought the punishment made the student angry as he viewed it negatively. After the punishment was instituted, the student started to have mood swings, withdrew from interacting with other students, and slept during the lessons. “*What I remember is that the other day during Tsonga period the deceased failed to present the assignment which the lady teacher gave us. The lady teacher told the deceased that it was a long time that she was following him to do the assignment and that he did not, she then took him to Mr. “Soza” (the deputy principal). The deceased was angry because this person gave him a punishment to pick the concrete at the playground for the whole month. The punishment made him furious and he thought teachers do not like him and every time something is wrong they point fingers at him”,* said a female peer and former class representative.

The participants in one of the completers’ groups mentioned that when students attempt suicide they do not want to die, but wish to communicate their needs to their parents. They further stated that some parents do not give their children attention at home neither time to find out what their children really want. This result in the child doing something to scare the parents hoping that after the scaring behaviour hopefully their needs will be met. Unfortunately, their actions resulted in completed suicide. This was the view of some students who indicated that the student needed a shoulder to cry on. *“He was crying out for help. You find that they did not give him that attention at home”,* said a female student*.*

In one of the groups a participant thought there was an evil spirit behind the peer’s death because it happened unexpectedly and without warning signs. The student believed that the death was a curse brought to their peer *“When I analyze what the principal explained to us with regard to her death I think it was a curse directed to her”,* said a female student. The pressure of school work was also a reason some students in three of the groups thought contributed to their peers’ suicide. Students leave their home in the morning and go back around five or six in the evening, depending on the distance as some students stay far from the school. Students are expected to be at school the whole day and on reaching home they have to do their assignments, prepare for school tests and at the same time have to cook and clean the house. Such a stressful environment full of activities with no rest period was seen by some students in one of the groups to contribute to the students’ suicide attempts. Some viewed a dismissal from attending school to contribute to the suicide. This was directed to a student who was recently dismissed from the school due to poor performance. The students stated that this could cause stress to this student as there was no school to accept him at that time of the year, secondly students thought that scolding by his parents could have add more stress on him, contributing to the decision to commit suicide.

The participants stated various factors they perceived to have caused the peer to attempted or complete suicide.

### Perceived available resources

Participants in all the groups mentioned that there were no services available at school to assist suicidal students and survivors of suicide, that is, suicide attempters, students, teachers and administrative staff affected by the suicide of the peer. Participants also expressed that the school is not concerned about their social life such as participating in sports activities, but the school concentrates on producing good Matric results by giving attention to good performing students, and ignore the slow learners *“We don’t have such services here at school. Actually the focus of our school is education. What they normally do they inform students during the assembly period that such an incident occurred, that is all they do. They won’t even try to assist in any way. Our school focuses on education only, other activities like sports are not of interest to them; as long as you get 80% in Maths”,* said a female student, from a private school.

### Views regarding prevention of suicide and suicide attempts

The students brought in several ideas during the discussions that they thought could help to reduce the scourge. A suggestion was made of organising a forum where students meet with an expert in the area of suicide to discuss issues affecting them. Students pointed out that such service should reach them not only if a student has completed suicide, as it was the case now. They gave an example of people who came after the death of their peer to offer some help like the priests and some non-government organisations, but ceased coming before a year after the death of a peer. Students saw people with expert knowledge of adolescents as relevant in addressing the students’ problems. A female student said: *“I think people need to talk more to the youth on this issue. People are just re-active. You only hear people trying to talk to the youth when a suicide has taken place. Other than such a situation you won’t hear people talking to the youth about stuff like that. I feel a person should be deployed to talk to the youth or in church at least once a month. Here you only find that they came to talk to us after he had committed suicide. They came and said if you have a problem come just now. I think it was not easy for people to respond. Let them try to reach out to us. At least there should be a forum in which the youth meet for talks at least once in a month”.*

Participants whose peers attempted suicide mentioned that it was high time for parents to learn to talk with their children politely*” Parents should not be harsh to their children when they made mistakes. They should not tell them to die so that they can have peace”* said a female peer.

One participant indicated that she was not in favour of people selling drugs because these contribute to suicide of young people. *“According to me people should not sell tobacco and drugs like Nyaope (mixture of dagga, cocaine and other intoxicating substances). Those who sell these drugs should be arrested”,* said a female student*.*

The focus groups of students with a peer who committed suicide had different views concerning the need for services than the groups with a peer who attempted suicide. The focus group discussions with students whose peers completed suicide view an expert as a solution in addressing young people’ problems in a forum, whereas the discussions with students of suicide attempters focused on the parents’ attitude that had to change.

## Discussion

In the present study, we aimed to investigate the experiences of high school students in Limpopo province whose peer attempted or completed suicide to understand their knowledge of and experiences with suicidal behaviour, and thus identify target areas for the development of a school-based suicide prevention programme in Limpopo. The methods used in the six cases identified in the present study were overdose of non-prescribed medication by two attempters while the victims for completed suicide all used hanging. The findings indicated that suicide attempts and the completed suicide of peers impacted the students’ emotional wellbeing differently. Those whose friends *attempted suicide* appeared less affected, and none of them cried during the interview, though they mentioned that they did cry when they first heard about the attempt due to uncertainty of the outcome. This reaction could have been due to the fact that the suicide attempters had the opportunity to explain the cause of their attempts which may have eased the guilt-feeling of their peers. On the contrary, the groups whose friend *completed suicide* were full of sadness and anger as demonstrated by their crying; wishing to have super natural powers of communicating with ghosts, and blaming themselves for the friend’s death. Unexpected deaths frequently produce initial defense mechanisms of shock, numbness, and denial. All of these reactions seem to happen when the death is a suicide. The feelings of anger among survivors is supported by Conroy [27] who found that survivors experience great anger either at themselves, the deceased or other people following a completed suicide. It can be argued that such behaviour could have resulted because they were not counseled following the suicide of the peer.

Participants reported that some peers who completed suicide displayed changes in behaviour some weeks or days before their suicide. In fact they seemed to bid farewell to their friends. The changes in behaviours included, sleeping during lessons, talking about death, withdrawing from social interactions, exhibiting mood changes, truancy, and writing messages on “mixit”. A few participants in each group reported that it was difficult to predict that the student was suicidal. The possible explanation for the different reported behaviour before the suicide attempts or completed suicides could be a hindsight bias, because after reviewing the suicide acts, some peers believed they observed the unusual behaviour displayed by the deceased students. It could be true that the classmates and peers could have identified such behaviours but were not sure what it meant due lack of knowledge of warning signs for suicidal behaviour. A study by Fisher [28] demonstrated that teens who attempted or completed suicide showed warning signs in advance, such as, talks about committing suicide, had trouble eating or sleeping, experienced drastic changes in behaviour, withdrew from friends or social activities, which seem similar to those displayed by the victims in the present study. Fisher also reports other warning signs, such as giving away prized possessions, having attempted suicide before, taking of unnecessary risks, losing of interest in his or her personal appearance, and increase in use of alcohol or drugs, but these signs were not reported in the present study [28].

One factor that was seen to be relevant for suicide attempts was a lack of communication between the student and family members, which was perceived to cause stress on the side of the student. A communication problem may mean that the student’s needs are not communicated to the parent, thus will be unmet. It may also denote lack of support from parents. This finding is supported by Shilubane et al. [16] and Zhang et al. [29] who found relationship problems as a determinant of suicide attempt and completed suicide among adolescents. Furthermore, Kerr et al. [11] and Shilubane, et al. [16, 17] found that poor family support was associated with suicidal ideation and suicide attempts among adolescents.

The student’s inability to cope with the school demands was thought to have contributed to their peers’ actions of committing suicide. The adolescent stage is a critical stage that comes with more demands and therefore requires support from both the teachers and the parents. If support is not provided in such a stressful situation, the adolescent may easily be influenced by depressed and suicidal peer and commit suicide as the result of copycat effects within their peer support system. This finding is supported by other studies that found adolescence as a stressful developmental phase in which young people with limited coping skills struggle to deal with suicidal ideation [30, 31].

Participants had a view that students do not want to be corrected by teachers and parents for their wrong doing and if corrected by somebody who is not the parent, they take it negatively. The South African Government does not allow teachers to use corporal punishment as a way of correcting wrong act by the students. It is stated that for wrong doings other measures have to be employed, though such measures have not been stipulated. It is not clearly stated which methods should be used to control children’s behaviour, and some teachers still use punishment to correct wrong behaviour. Punishment is often associated with child abuse and it is not surprising to find students committing suicide when punishment is instituted on them [32].

The suicide attempt and the completed suicide by peers was viewed by the peers as a way of drawing the attention of parents or significant others, not that they wanted to die. As perceived by the peers, they were looking for attention and trying to alert their parents, this attention seeking somehow ended in tragedy for some students. This behaviour is supported by Roen, Scourfield and McDermott [33] who found suicide attempt as a cry for connection, while Tam and colleagues [34] found that most teenagers do not attempt suicide to harm them-selves but want to make people around them understand how desperate they are, or want to teach others a lesson. Students should be taught appropriate ways of communicating their needs to their parents and those around. This could be done through school lessons by teachers as part of the suicide prevention program. Furthermore, the perceived causes for suicide attempts were different from causes for completed suicides, one can speculate whether their impacts are different or not, which warrants further research.

It became clear during the discussions that there are no services available at the schools where the participants were recruited from. It should be noted that psychological help is provided mostly to suicide attempters while the affected bystanders are ignored. Before the victim is discharged from a hospital following a suicide attempt, an arrangement with the psychologist is made for counseling services. It is clear that fellow students did not receive such services and this could have impacted on their psychological wellbeing. It is therefore not surprising to find that some students were preoccupied with suicide after a suicide of their peer, which could be because no one paid attention to their affected minds. As South Africa is a middle-income country with mental health care services being scarce and mostly under-resourced, development of life skills and healthy decision making of high school students through evidence-based suicide prevention programs at school could assist in the reduction of suicide rates among high school students. These programs have demonstrated to reduce the risk of suicide among the youth [35, 36].

The participants brought their opinions which they thought could help to curb this scourge, such as a forum conducted by an experienced person where young people can meet and share their experiences. They emphasized the need for such meetings in the sense that some parents do not discuss important life issues with their children, especially not on sexual matters irrespective of Turnball, van Wersch and van Schaik [37] who demonstrated in their review of studies into parental involvement in sex education that parents are the primary sexual educators. It is imperative that parents made aware in this regard. A meeting between students and those people who once experienced crisis in their lives but managed to overcome was regarded as worth sharing with students. Some participants’ perceived tobacco and drug use to be associated with suicide and mentioned the need for prevention of substance abuse at school. Previous studies among young people also found substance abuse to be correlated with suicidal behaviour [26, 38–41].

## Conclusion

### Recommendations and conclusion

Early identification of warning signs by fellow students and referral to professional help might prevent suicidal behaviour among adolescents. In the lives of many high school students peers are the most important source of social support [25]. Fellow students are often the first to know of a peer’s mental health problems or suicidal thoughts. However, peers and classmates are also frequently unsure what to do with this information as was also found in the present study. Given the role peers play in the lives of youngsters, they could be an important target group for suicide prevention programs.

The present study is part of a series of studies (Psychosocial determinants of suicide attempts among black South African adolescents: a qualitative analysis, Psychosocial Correlates of Suicidal Ideation in Rural South African Adolescents, Suicide and related health risk behaviours among school learners in South Africa: results from the 2002 and 2008 national youth risk behaviour surveys, Explaining suicide in South African adolescents: psychosocial and environmental precursors) that provide input for the development of a comprehensive school-based suicide prevention program in Limpopo province, targeting students, teachers, school management, and school health staff to promote early identification of suicidal cases and install social support systems. These studies comprise the first step (needs assessment) in the systematic development of the program according to Intervention Mapping [42].

Despite the limited generalizability of the present study because of the small sample and the specific geographic location under study, the findings point at the need to introduce mental health and suicide as topics in the existing curriculum to help students in identifying suicidal peers and providing support through informing or referral to relevant people. The teaching on mental well-being and suicide may for example take the format of health talks and awareness campaigns organized by school health nurses. This component could exist next to training programs for teachers to increase their knowledge of warning signs for suicidal behaviour and appropriate responses when confronted with a suicidal student (see Shilubane et al., under review) [43]. In addition, counseling services are to be installed within the school environment to provide pre-counselling for students with suicidal thoughts as well as post suicide counselling for peers and class mates to lower the negative impact of the event on personal wellbeing and avoid for example cluster suicides. This may also reduce the students’ suicide rate especially when everybody knows where to go when peers have suicidal thoughts. In addition, counseling services may help to address stressors within the home environment such as conflicts arising from family relationships and school environment like peer pressure and school work. Research on suicide prevention program development is needed, thereby incorporating possibilities to distinguish between instances of suicide completion and suicide attempt as peers react differently in these instances and therefore may need different interventions.
